# A Random Forest Sub-Golgi Protein Classifier Optimized via Dipeptide and Amino Acid Composition Features

**DOI:** 10.3389/fbioe.2019.00215

**Published:** 2019-09-04

**Authors:** Zhibin Lv, Shunshan Jin, Hui Ding, Quan Zou

**Affiliations:** ^1^Institute of Fundamental and Frontier Sciences, University of Electronic Science and Technology of China, Chengdu, China; ^2^Department of Neurology, Heilongjiang Province Land Reclamation Headquarters General Hospital, Harbin, China; ^3^Center for Informational Biology, University of Electronic Science and Technology of China, Chengdu, China

**Keywords:** random forests, sub-Golgi protein classifier, ANOVA feature selection, split amino acid composition, k-gap dipeptide, synthetic minority over-sampling

## Abstract

To gain insight into the malfunction of the Golgi apparatus and its relationship to various genetic and neurodegenerative diseases, the identification of sub-Golgi proteins, both cis-Golgi and trans-Golgi proteins, is of great significance. In this study, a state-of-art random forests sub-Golgi protein classifier, rfGPT, was developed. The rfGPT used 2-gap dipeptide and split amino acid composition for the feature vectors and was combined with the synthetic minority over-sampling technique (SMOTE) and an analysis of variance (ANOVA) feature selection method. The rfGPT was trained on a sub-Golgi protein sequence data set (137 sequences), with sequence identity less than 25%. For the optimal rfGPT classifier with 93 features, the accuracy (ACC) was 90.5%; the Matthews correlation coefficient (MCC) was 0.811; the sensitivity (Sn) was 92.6%; and the specificity (Sp) was 88.4%. The independent testing scores for the rfGPT were ACC = 90.6%; MCC = 0.696; Sn = 96.1%; and Sp = 69.2%. Although the independent testing accuracy was 4.4% lower than that for the best reported sub-Golgi classifier trained on a data set with 40% sequence identity (304 sequences), the rfGPT is currently the top sub-Golgi protein predictor utilizing feature vectors without any position-specific scoring matrix and its derivative features. Therefore, the rfGPT is a more practical tool, because no sequence alignment is required with tens of millions of protein sequences. To date, the rfGPT is the Golgi classifier with the best independent testing scores, optimized by training on smaller benchmark data sets. Feature importance analysis proves that the non-polar and aliphatic residues composition, the (aromatic residues) + (non-polar, aliphatic residues) dipeptide and aromatic residues composition between NH2-termial and COOH-terminal of protein sequences are the three top biological features for distinguishing the sub-Golgi proteins.

## Introduction

The Golgi apparatus (GA) is an important organelle in eukaryotic cells, because lipids and different types of proteins are modified, packaged, and transported in vesicles to different destinations (Rhee et al., 2005). The GA comprises three main parts (Xu and Esko, 2009): cis-Golgi, medial, and trans-Golgi. The cis-Golgi receives proteins and then delivers them to the medial section for protein biosynthesis. The trans-Golgi releases the biosynthesized proteins from the medial section. The proteins in the cis-region of the GA are called cis-Golgi proteins, whereas trans-Golgi proteins are in the trans-Golgi part (Pfeffer, 2001).

Malfunction of the GA can disrupt protein biosynthesis in the medial part, which can lead to neurodegenerative diseases, such Parkinson's (Fujita et al., 2006; Yang J. et al., 2016) and Alzheimer's (Gonatas et al., 1998; Yang et al., 2015). A key step in the understanding of GA function is to determine whether a protein is a sub-Golgi protein (cis-Golgi or trans-Golgi). Such determinations will improve comprehension of the mechanisms for GA dysfunction and provide clues for disease treatment and more effective drug research and development (Gunther et al., 2018).

In the past few years, several protein subcellular locations and protein type prediction tools, including sub-Golgi protein identification tools (Teasdale and Yuan, 2002; Van Dijk et al., 2008; Chou et al., 2010; Ding et al., 2011, 2013; Jiao et al., 2014; Lin et al., 2014; Nikolovski et al., 2014; Jiao and Du, 2016a,b; Yang R. et al., 2016; Ahmad et al., 2017; Wang et al., 2017; Rahman et al., 2018; Ahmad and Hayat, 2019; Wuritu et al., 2019), have been developed using various machine learning algorithms, including increment diversity Mahalanobis discriminant (IDMD) (Ding et al., 2011), support vector machine (SVM) (Ding et al., 2013, 2017; Jiao et al., 2014; Lin et al., 2014; Jiao and Du, 2016a,b), random forest (RF) (Ding et al., 2016a,b; Yang R. et al., 2016; Yu et al., 2017; Liu et al., 2018), and K nearest neighbor algorithm (KNN) (Ahmad et al., 2017; Ahmad and Hayat, 2019), among others. To generate feature vectors for sub-Golgi protein identification, protein amino acid composition (AAC) (Rahman et al., 2018), k-gapped dipeptide composition (k-gapDC) (Ding et al., 2011, 2013), pseudo amino acid composition (PseAAC) (Jiao et al., 2014; Liu et al., 2015), and protein sequences evolutionary information (e.g., position-specific scoring matrix, PSSM) and their derivative features (Yang et al., 2014; Jiao and Du, 2016a,b; Yang R. et al., 2016; Ahmad et al., 2017; Rahman et al., 2018) have been used. Because the extensively used training benchmark data sets (Ding et al., 2013; Yang R. et al., 2016) are unbalanced in sub-Golgi protein classes, a synthetic minority over-sampling technique (SMOTE) has been adopted to obtain class-balanced data sets for training (Yang R. et al., 2016; Ahmad et al., 2017; Wan et al., 2017; Rahman et al., 2018; Ahmad and Hayat, 2019). Diversified feature selection methods, including analysis of variance (ANOVA) (Ding et al., 2013; Jiao and Du, 2016a), minimal redundancy-maximal relevance (mRMR) (Jiao and Du, 2016b; Wang S. P. et al., 2018), maximum relevance-maximum distance (MRMD) (Zou et al., 2016a,b), RF/Wrapper (Pan et al., 2018; Rahman et al., 2018), multi-voting for feature selection (Ahmad and Hayat, 2019), and lasso (Liu et al., 2016), among others, have been used to remove redundant features and improve the prediction accuracy with as few features as possible (Yu et al., 2016; Zhu et al., 2017, 2018; Kuang et al., 2018; Wang H. et al., 2018).

Two widely used benchmark-training data sets have resulted in different optimization models with various independent testing prediction scores. For the benchmark data set of Ding (137 sequences with 25% sequence identity; Ding et al., 2013), Jiao and Du (2016b) applied 49-dimensional features of positional-specific physicochemical properties (PSPCP, a derived feature from PSSM) to train their best SVM model. They achieved jackknife cross-validation results with accuracy (ACC) of 91.2%; Matthew correlation coefficient (MCC) of 0.793; sensitivity (Sn) of 99.0%; and specificity (Sp) of 73.8%, whereas the independent prediction accuracy of their classifier was 87.1%. The best predictor built on the benchmark data set of Yang (304 sequences with 40% sequence identity) (Yang R. et al., 2016) was developed by Ahmad and Hayat (2019). They carefully selected 180-dimensional features from the combined features of split amino acid composition (SAAC), 3-gap dipeptide composition, and PSSM with its derivative features to obtain a designed KNN classifier with good jackknife cross-validation scores (ACC = 94.9%; MCC = 0.90; Sn = 97.2%; Sp = 92.6%) and good independent testing scores (ACC = 94.0%; MCC = 0.84; Sn = 81.5%; Sp = 96.9%).

To our best knowledge, all high-profile sub-Golgi protein predictors trained on either benchmark data sets are constructed on the basis of a PSSM and its derived feature vectors, whose acquisition requires the use of a position-specific iterative basic local alignment search tool to align sub-Golgi protein sequences with a protein database (Jiao and Du, 2016a,b; Rahman et al., 2018; Ahmad and Hayat, 2019). Then, a secondary data transformation is performed (Altschul et al., 1997) in which data are usually converted into a 20 by 20 matrix with average values in each feature dimension (Jiao and Du, 2016a,b; Yang R. et al., 2016; Ahmad et al., 2017; Rahman et al., 2018). The sequence alignment is typically time-consuming, particularly when the protein database for alignment is large and the computing power is limited.

In this paper, instead of using PSSM and its derived features, the focus was on constructing an efficient sub-Golgi protein RF classifier, namely rfGPT, based only on amino acid and dipeptide composition-based feature vectors. Related studies (Li et al., 2016; Luo et al., 2016; Tang et al., 2018; Zhang et al., 2018a,b) have demonstrated the effectiveness of composition and dipeptide and amino acid composition-based features for solving bioinformatics problems. The rfGPT with 55-dimensional features of 2-gap dipeptide composition attained better jackknife cross-validation scores (ACC = 91.1%; MCC = 0.823; Sn = 87.4%; Sp = 94.7%) and better independent testing results (ACC = 89.1%; MCC = 0.631; Sn = 53.8%; Sp = 98.0%) than those classifiers trained on the same data set (Ding et al., 2013; Jiao and Du, 2016a,b). Therefore, to date, the rfGPT is the best sub-Golgi predictor trained from the benchmark data set of Ding via SMOTE (Ding et al., 2013). For further improvement of the rfGPT, 59 2-gap dipeptide composition features selected through ANOVA technology were fused with SAAC features to form 119 new dimensional features, which were then secondarily selected via ANOVA for rfGPT optimization. Ultimately, the rfGPT with 93 dimensional features [59 2-gap dipeptide composition (DC) sub-features plus 34 SAAC sub-features] was the best predictor, with jackknife cross-validation scores of ACC = 90.5%; MCC = 0.811; Sn = 92.6%; and Sp = 88.4%, and independent test scores of ACC = 90.6%; MCC = 0.696; Sn = 96.1%; and Sp = 69.2%.

## Materials and Methods

### Data Sets

To train models for sub-Golgi protein identification, two benchmark-training data sets are widely used. One data set, D1 in this text, was constructed by Ding et al. (2013), and the other, D2 in this text, was constructed by Yang R. et al. (2016). Before D1 was developed, Ding et al. constructed a smaller data set (D0) which was used once and never used again (Ding et al., 2011). In this work, the data set D1 was downloaded from http://lin-group.cn/server/SubGolgi/data and used to train the sub-Golgi protein classifier. The D1 data set consisted of 137 Golgi-resident protein sequences, with 42 cis-Golgi and 95 trans-Golgi proteins. The D1 data set was selected for model training primarily because the sequence identity was <25%. Thus, the D1 data set contained less sequence noise and redundancy than the D2 data set.

For testing the optimized model, an independent data set D3 provided by Ding et al. (2013) was applied. The D3 data set has been adopted by most of the key researchers in previously reported sub-Golgi predictors (Ding et al., 2013; Jiao and Du, 2016b; Yang R. et al., 2016; Ahmad et al., 2017; Rahman et al., 2018; Ahmad and Hayat, 2019). The D3 data set is generally used only for independent testing and contains 64 test sequences, including 13 cis-Golgi and 51 trans-Golgi protein sequences. The D3 data set is available at http://lin-group.cn/server/SubGolgi/data.

### Modeling Overview

**Figure 1:**
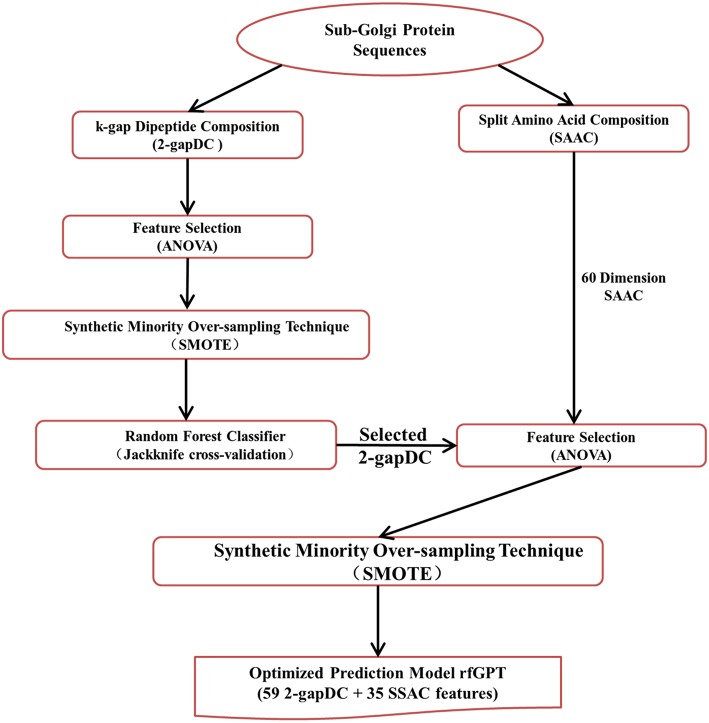
Modeling framework of the state-of-art random forests sub-Golgi protein classifier. ANOVA: analysis of variance.

The entire rfGPT modeling process is illustrated in Figure 1. Compared with previous predictors, the major difference of the rfGPT used in this study was that only extracted features from amino acid and dipeptide composition were used. In this study, the 2-gapped dipeptide composition profile and SAAC were adopted. Ding et al. (2013) verified the validity of the 2-gapped dipeptide composition profile for sub-Golgi prediction. The SAAC considers that the location of a Golgi protein is related to the composition of amino acid residues at the N-terminal and C-terminal of a protein sequence (Paulson and Colley, 1989). As shown in Figure 1, the 400 dimensions (400D) 2-gapDC features extracted from D1 were used to generate a class-balanced data set via ANOVA and SMOTE, which was then fed into a RF model for optimization and estimation by jackknife cross-validation and independent testing. In this step, an optimized prediction model was sought, whose selected features were then combined with the SAAC features as new features of a new model for further optimization. After the secondary feature selection via ANOVA and SMOTE, the new optimal model was evaluated through jackknife cross-validation and independent testing.

### Feature Extraction

The methods for feature extraction used for sub-Golgi classification are divided into three categories: (1) amino acid and peptide composition and their derived features; (2) PSSM and its derived features; and (3) features combined with amino acid residue physical and chemical properties. In this research, the derived features of category 1 were adopted because they are simple and convenient for feature extraction, namely, to calculate the frequency of peptide and amino acid components. The following two AAC features were adopted.

#### k-Gapped Dipeptides Composition

In general, the composition of adjacent dipeptides can only reflect the short-range structure of the protein sequence. The dipeptide composition in the larger interval may better reflect the tertiary structure of the protein. In biology, interval residues are more important than adjacent residues. Especially in some common structures, such as helices and plates, two non-adjacent residues are joined by hydrogen bonds (Lin et al., 2015; Wang et al., 2019). The k-gap dipeptides composition (k-gapDC) is an indirect mathematical description of the biological significance, which has been extensively utilized for sub-Golgi protein classification and other bioinformatics fields (Xu et al., 2018; Agrawal et al., 2019; Akbar et al., 2019; Wang et al., 2019). For the k-gapDC, the frequency of a dipeptide separated by k positions is determined, which is then divided by the total number of k-gapped dipeptides; thus, a protein sequence is transformed into a 400D feature vector. The 2-gapDC features were utilized in this work.

#### Split Amino Acid Composition

It has been proved that the N-terminal and C-terminal of protein sequences can act as signal-anchor domains for subcellular locations, e.g., glycosyltransferases all have a short NH2-terminalcytoplasmic tail, a 16-20-amino acid signal-anchor domain, and an extended stem region which is followed by the large COOH-terminal catalytic domain (Paulson and Colley, 1989). Another example is that lysine at position 329 within a C-terminal dilysine motif is crucial for the endoplasmic reticulum localization of human SLC35B4 (Bazan et al., 2018). All of these inspire us to used split amino acid composition for sub-Golgi protein identification. The split amino acid composition was proposed by Chou (Chou and Shen, 2007), which converts variable-length protein sequences into fixed-length amino acids for feature representation. In SAAC, a protein sequence is initially segmented into different parts, and then the amino acid frequency of each independent part is calculated. In the current work, the protein sequences were split into three segments: 30 N-terminal residues, 30 C-terminal residues, and the intermediate-block residues, which are the sequences between N-terminal and C-terminal parts. A 60D feature vector was obtained from the SAAC instead of the traditional 20D amino acid component. The details of the SAAC feature extraction are described as follows. Considering the length of protein sequence L and the three segments [NSeg (N-terminal), ISeg (intermediate block), and CSeg (C-terminal)] with the lengths Xn, L – Xn – Xc, and Xc (Xn = Xc = 30), respectively, the SAAC feature vector [*f*_1_, *f*_2_, ··· , *f*_60_] is generated by the following formulas:

- $f_{i}=\frac{N\left(AA_{i}\right)}{X_{n}},\;i=\;1,2,\ldots ,20$
- $f_{i}=\frac{N\left(AA_{i}\right)}{L-X_{n}-X_{c}},\;i=\;21,22,\ldots ,40$
- $f_{i}=\frac{N\left(AA_{i}\right)}{X_{c}},\;i=\;41,42,\ldots ,60$
  *AA* : amino acid residue;
  *N*(*AA*) : the numbers of AA in different segments.
  *L*: the length of protein sequence;
  *X*_*n*_: the residues numbers of N-terminal segments;
  *X*_*c*_: the residues numbers of C-terminal segments.
  *f*_*i*_: the ith SAAC feature vector element, it is one of the 20 amino acid residue frequency in a segment.

### Feature Selection

Feature selection is conducted to remove redundant information and to overcome over-fitting in machine learning modeling. A variety of feature selection techniques (Ding et al., 2013; Jiao et al., 2014; Zeng et al., 2015, 2016, 2018; Jiao and Du, 2016a,b; Yang R. et al., 2016; Ahmad et al., 2017; Rahman et al., 2018; Ahmad and Hayat, 2019; Liu Y. et al., 2019; Zhang X. et al., 2019) have been important for sub-Golgi protein identification and for other areas of bioinformatics. ANOVA ranks the importance of features in terms of the ratio of the variance of data within a category to the variance between categories. The larger the value of the ratio is, the more important the feature is. The details for the use of ANOVA as a feature selection technique have been presented previously (Ding et al., 2013; Jiao and Du, 2016a) and are not repeated here. In this study, the ANOVA module from the famous Scikit-learn machine learning tool kit was used for feature selection (https://scikit-learn.org/).

### Synthetic Minority Over-sampling Technique

The D1 benchmark data set is imbalanced, with the cis-Golgi protein and trans-Golgi protein sequences ratio of 0.44. Such an imbalance has a significant impact on the acceptability of the application, because the classifiers can be overly suitable for the majority classes. In this case, the prediction accuracy may seem high, but the results may be unacceptable, as minority groups may be completely/partially ignored. To solve this problem, the very effective SMOTE was proposed by Chawla et al. (2002). SMOTE helps to balance unbalanced data sets by creating “synthetic” minority class examples rather than by oversampling with replacement, and is employed by various sub-Golgi classifiers trained on benchmark data set D2 (Yang R. et al., 2016; Ahmad et al., 2017; Rahman et al., 2018; Ahmad and Hayat, 2019). As this manuscript was prepared, the use of SMOTE with benchmark data set D1 had not yet been reported. In this research, the SMOTE module implemented was from http://imbalanced-learn.org.

### Evaluation Metrics

#### Testing Methods

The jackknife cross-validation is a leave-one-out cross-validation method for testing the efficiency of protein classification (Chou and Shen, 2006) and is executed in the following steps. A training data set with T items is separated into two parts. For each run, one part consists of T−1 item for model training, and the remaining part contains one item for testing. This process is repeated T times, and all the items sampled in the training data set act as a testing sample only once. Jackknife cross-validation is a time-consuming method, particularly for large data sets, but the method is robust with small variance. In this article, the benchmark data set D1 collected by Ding et al. (2013) was used for the jackknife cross-validation.

In independent testing, a completely different data set from the training data set is used to evaluate the trained model. Once the model is built with the training data set, tests are performed on the independent data set to evaluate the model. In this article, the independent data set D3 collected by Ding et al. (2013) was used for model performance evaluation.

#### Performance Metrics

Four standard metrics were used to evaluate the proposed models: ACC, Sn, Sp, and MCC. The metrics are previously described (Wei et al., 2017a,b; Chen et al., 2018; Su et al., 2018; Feng et al., 2019; Zhang S. et al., 2019) and were calculated as follows:

- $ACC=\frac{TP+TN}{TP+TN+FP+FN}$
- $S_{n}=\frac{TP}{TP+FN}$
- $S_{p}=\frac{TN}{TN+FP}$
- $MCC=\frac{TP\;\times \;TN-FP\;\times \;FN}{\sqrt{\left(TP+FP\right)\;\times \;\left(TN+FN\right)\;\times \;\left(TP+FN\right)\;\times \;\left(TN+FP\right)}}$

where TP is a true positive, TN is a true negative, FP is a false positive, and FN is a false negative.

### Classifier

Support vector machine (SVM) (Ding et al., 2011, 2013; Feng et al., 2013; Lin et al., 2014; Jiao and Du, 2016a,b; Zeng et al., 2017; Rahman et al., 2018; Chen et al., 2019; Dao et al., 2019; Liu B. et al., 2019), K-nearest neighbor (KNN) (Ahmad et al., 2017; Ahmad and Hayat, 2019), and random forests (RF) (Yang R. et al., 2016; Pan et al., 2017; Ru et al., 2019; Su et al., 2019; Zheng et al., 2019) classifiers have been used to identify sub-Golgi proteins and for other fields. In this study, RF was selected for modeling because it is a powerful machine-learning tool and facilitates analysis of feature importance. Previously, Yang R. et al. (2016) selected 55 features from composite features (3-gapDC + PSSM derived features) to optimize their random forest classifier. The jackknife cross-validation scores using data set D2 were ACC = 88.5%; MCC = 0.765; Sn = 88.9%; and Sp = 88.0%, and for the independent testing, the scores were ACC = 93.8%; MCC = 0.821; Sn = 92.3%; and Sp = 94.1% (Yang R. et al., 2016). However, those results are somewhat confusing, because other sub-Golgi predictors have lower independent test scores than those for the jackknife cross-validation. To date, no sub-Golgi RF predictor has been trained from benchmark data set D1. In this study, the random forest classification model in the Scikit-learn tool kit (https://scikit-learn.org/) was applied for the implementation, testing, and evaluation of the rfGPT classifier and for the analysis of feature importance.

## Results and Discussion

### Performance of Random Forests Classifier Without Feature Selection

Table 1 shows the performance of the rfGPT using various extracted features. In the models with the SMOTE technique, the cross-validation scores improved remarkably for ACC, MCC, Sn, and Sp. For example, based on 460D SAAC + 2-gapDC features and SMOTE, the scores of the rfGPT were ACC = 90.5%; MCC = 0.817; Sn = 96.8%; and Sp = 84.2%, which were increases of 20, 132, 44, 2.2, and 171.6%, respectively, compared with the rfGPT without SMOTE. Although the SMOTE technique does improve the recognition rate of minority classes, the accuracy of the independent testing for the rfGPT with diverse features ranged from 78.1 to 82.8%, with little improvement with SMOTE (Table 1). For the other metrics (MCC, Sn, Sp), the case was the same. Thus, other techniques are needed to improve the generalization prediction model. In this paper, to obtain a better rfGPT with fewer features, ANOVA feature selection was used to eliminate redundant features.

**Table 1:** Jackknife cross-validation and independent testing results after training on the benchmark data set D1 without feature selection.

**Feature(D)**	**SMOTE (Y/N)**	**Jackknife cross-validation**				**Independent testing**			
		**ACC**	**MCC**	**Sn**	**Sp**	**ACC**	**MCC**	**Sn**	**Sp**
2-gapDC(400)	N	74.5%	0.326	94.7%	28.6%	79.7%	0.318	90.2%	38.5%
SAAC(60)	N	69.3%	0.073	97.9%	4.8%	78.1%	−0.07	98.0%	0.0%
2-gapDC+SAAC(460)	N	75.2%	0.351	94.7%	31.0%	79.7%	0.237	94.1%	23.1%
2-gapDC(400)	Y	86.3%	0.743	96.8%	75.8%	82.8%	0.351	98.0%	23.1%
SAAC(60)	Y	87.9%	0.763	93.7%	82.1%	81.2%	0.388	90.2%	46.2%
SAAC+2-gapDC(460)	Y	90.5%	0.817	96.8%	84.2%	81.2%	0.287	96.1%	23.1%

### Classifier Optimizing via ANOVA Feature Selection

**Figure 2:**
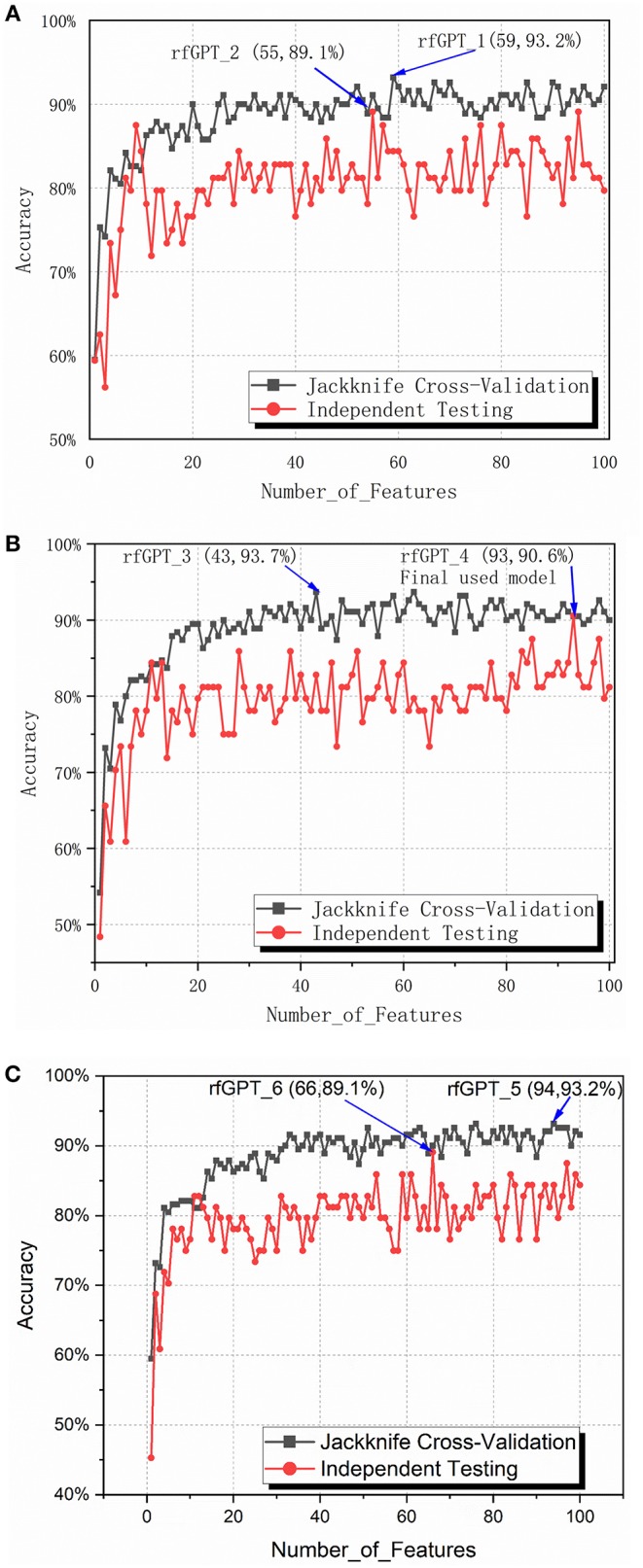
Jackknife cross-validation and independent testing accuracy of the random forest classifier with the number of features varied: **(A)** 2-gap dipeptide composition (2-gapDC) features **(B)** 59 selected 2-gapDC features + 60 split amino acid composition (SAAC) features, and **(C)** 55 selected 2-gapDC features + 60 SAAC features.

To obtain the optimized classifier, the ANOVA feature selection method was first conducted for 400 2-gapDC features. One hundred sub-data sets containing 1, 2, … and 100 2-gapDC features generated separately after ANOVA feature selection were used for training 100 corresponding RF classifiers. For all 100 classifiers, jackknife cross-validation and independence testing were conducted. Figure 2A shows the accuracy of the cross-validation and independent tests of the 100 classifiers with varying numbers of features. Except for the models with nine and ten selected features, the average accuracy of the jackknife cross-validation of the other models was higher than that of the independent test results. Based on the jackknife cross-validation, the best-trained model with the highest accuracy was the classifier with 59 selected features (rfGPT_1), whereas the classifier with 55 selected features (rfGPT_2) had the highest independent testing accuracy results.

The performance scores of both classifiers are listed in Table 2. The jackknife cross-validation scores of rfGPT_2 (ACC = 91.1%; MCC = 0.823; Sn = 94.7%; Sp = 87.4%) were slightly lower than those of rfGPT_1 (ACC = 93.2%; MCC = 86.4%; Sn = 94.7%; Sp = 91.6%). However, rfGPT_2 had the better predictive performance on the independent test sets with scores of ACC = 89.1%; MCC = 0.631; Sn = 98%; and Sp = 53.8%, which were as much as 5.6, 35, 8.3, 10, and 16% larger than the corresponding values of rfGPT_1 (ACC = 84.4%; MCC = 0.466; Sn = 94.1%; Sp = 46.2%). The 89.1% independent testing accuracy of rfGPT_2 was an increase of 2.2% compared with the best SVM sub-Golgi classifier (Jiao and Du, 2016b) trained on the same benchmark data set (D1). The accuracy of 93.2% for rfGPT_1 and 91.1% for rfGPT_2 from the jackknife cross-validations was an increase of 9.0 and 6.5%, respectively, compared with that of the RF classifier obtained by Yang et al. which was trained on benchmark data set D2 (Yang R. et al., 2016).

**Table 2:** The best evaluation scores from jackknife cross-validation and independent testing of different models with various feature types and feature numbers.

**Classifier**	**Features(D)**	**Jackknife cross-validation**				**Independent testing**			
		**ACC**	**MCC**	**Sn**	**Sp**	**ACC**	**MCC**	**Sn**	**Sp**
rfGPT_1	2-gapDC(59)	93.2%	0.864	94.7%	91.6%	84.4%	0.466	94.1%	46.2%
rfGPT_2	2-gapDC(55)	91.1%	0.823	94.7%	87.4%	89.1%	0.631	98.0%	53.8%
rfGPT_3	2-gapDC+SAAC(43)	93.7%	0.874	93.7%	93.7%	82.8%	0.484	88.2%	61.5%
rfGPT_4	2-gapDC+SAAC(93)	90.5%	0.811	92.6%	88.4%	90.6%	0.696	96.1%	69.2%
rfGPT_5	2-gapDC+SAAC(94)	93.2%	0.864	93.7%	92.7%	84.4%	0.546	88.2%	69.2%
rfGPT_6	2-gapDC+SAAC(66)	90.0%	0.800	89.5%	90.5%	89.1%	0.695	90.2%	84.6%

For further optimization, the 59 2-gapDC features of rfGPT_1 obtained in the previous step were combined with 60 SAAC features to form 119-dimensional (2-gapDC + SAAC) composite features, and then ANOVA was used to construct 100 data sets with selected 1, 2, ··· and 100 features for building 100 classifiers. The jackknife cross-validation and independent test results for these models are shown in Figure 2B and Table 2. For the cross-validation performance, classifier rfGPT_3 with 43 features was better than classifier rfGPT_4 with 93 features. However, for independent testing, the predictive metric of rfGPT_4 with ACC = 90.6%; MCC = 0.696; Sn = 96.1%; and Sp = 69.2% exceeded that of rfGPT_3 with ACC = 84.4%; MCC = 0.466; Sn = 88.2%; and Sp = 61.5%; the increases were 7.3%, 49, 8.3, 9.0, and 13%, respectively.

Optimization was also performed by combining the 55 2-gapDC features of rfGPT_2 with SAAC features to form 115-dimensional features for 100 new models with various features. The cross-validation and independent testing accuracy scores are revealed in Figure 2C. The scores for rfGPT_5 and rfGPT_6 are shown in Table 2. The independent accuracy of both models was inferior to that of rfGPT_4 (Table 2).

Because most cross-validation and independent testing scores of the classifier rfGPT_4 were superior to those of other models in Table 2, rfGPT_4 was designated as the final sub-Golgi model for prediction.

### Feature Importance Analysis

**Figure 3:**
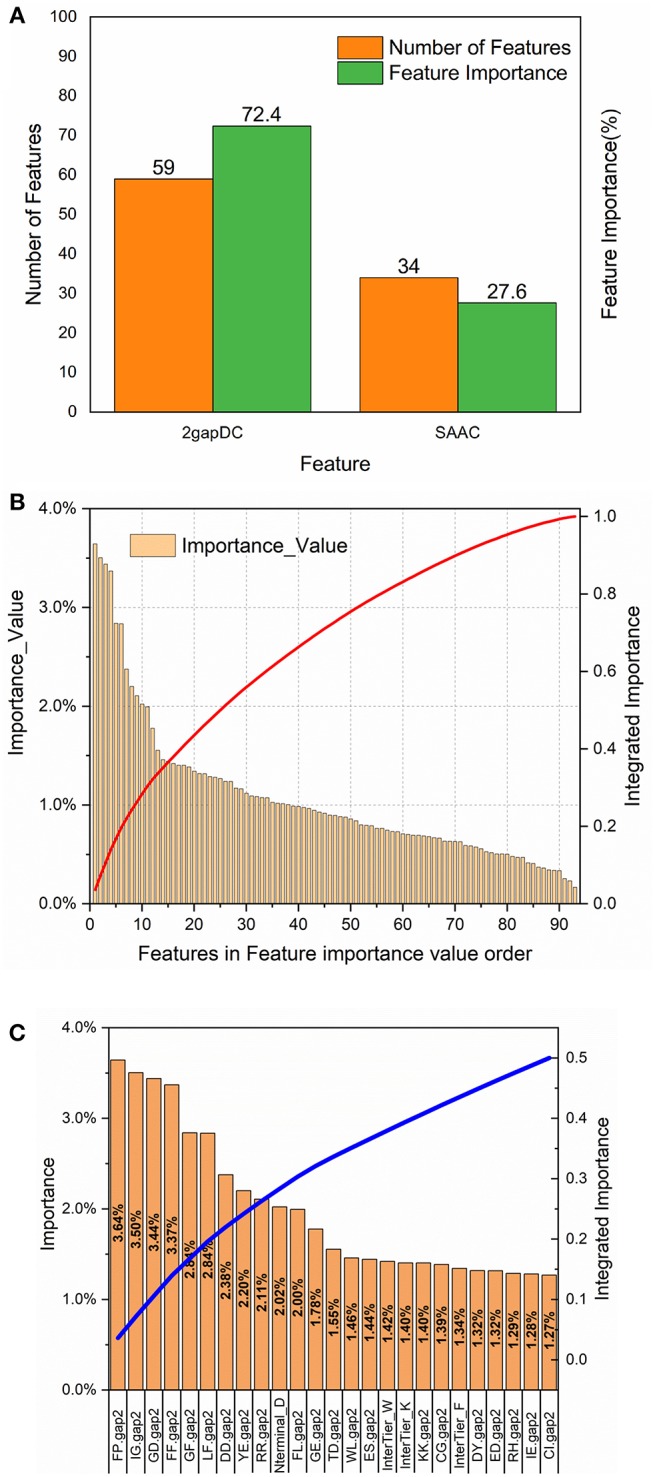
Feature importance analysis of random forests sub-Golgi classifier, rfGPT _4: **(A)** importance of feature types **(B)** the ranking orders of 93 features for rfGPT_4 and their integrated importance (red line), and **(C)** the importance of the top 25 features, which accounted for 50% of the integrated importance (blue line). The A_1_A_2_.gap2 means the composition of dipeptide A1A2. A1 or A2 is one of the 20 amino acid residues. Nterminal_D means the composition of amino acid residues D (aspartate) in NH_2_-terminal of protein sequence. InterTier_K, interTier_W, and interTier_F mean K(lysine), W(tryptophan), and F(phenylalanine) amino acid residues composition of the inter-tier between NH_2_-terminal and COOH-terminal of protein sequence.

To analyze the importance of the features selected for rfGPT_4, the feature importance function of the Scikit-learn RF model was exploited (Figure 3). As shown in Figure 3A, 59 2-gapDC features and 34 SAAC features were adopted in rfGPT_4, and their importance to the classification of Golgi proteins was 72.4 and 27.6%, respectively. Figure 3B shows the ranking of the 93 features by importance value and the cumulative importance score by importance value order. Among the combined features, the single feature importance was diverse and ranged from 0.16 to 3.64%. Figure 3C shows the importance order of the first 25 specific features, which accounted for 50% of the importance for the rfGPT. Only four of the top 25 features (which included 21 2-gapDC features and 4 SAAC features) had an importance value of more than 3% (Figure 3C).

To further analyze the feature bio-meaning, the feature importance values are assigned to different types of amino acid residues, that is aromatic residues, non-polar, and aliphatic residues, polar and non-charged residues, positively charged residues, and negatively charged residues. For instance, FP.gap2 feature as shown in Figure 3C means the composition frequency of dipeptide, which consists of F (phenylalanine) and P (proline) amino acid residence. The importance value 3.64% for FP.gap2 feature is divided by 2 to allocate 1.72% to aromatic residues type and non-polar and aliphatic residues type. Other features importance values are handled in the same way to assign importance value to five type amino acid residues (see Table S1). It finds out that the importance value of non-polar and aliphatic residues, aromatic residues, negatively charged residues, positively charged residues, polar, and non-charged residues are 30%, 24%, 21%, 13% and 12%, respectively. The non-polar and aliphatic property of amino acid residues plays the most critical role in sub-Golgi protein identification, and then the next is aromatic, negatively charged, positively charged, and polar and non-charged in turn. The importance values of the first three properties add up to 75%, so it concludes that to discriminate cis or trans sub-Golgi protein is mainly determined by the non-polar and aliphatic residues, aromatic residues, and negatively charged residues composition frequency.

For 2-gap DC features, the first three most important features are FP.gap2 (3.64%), IG.gap2 (3.50%), and GD.gap2 (3.44%), and five different residue types combined with each other generate 25 type dipeptides, whose feature importance values are listed in Figure 3C and Table S2. The (aromatic residues) + (non-polar, aliphatic residues) dipeptide, (non-polar, aliphatic residues) + (non-polar, aliphatic reduces residues) dipeptide and the (non-polar, aliphatic residues) + (aromatic residues) with the importance values as 8.54%, 8.18%, and 7.36%, respectively, are the top three important features for sub-Golgi classification.

For SAAC features, the protein sequence is segmented into three parts: N-terminal segment, C-terminal segment and the Interblock between N-terminal and C-terminal, whose amino acid composition frequency feature is labeled as Nterminal_A, Cterminal_A and InterTier_A (A represents one of the 20 amino acid residues; see Figure 3C and Table S3). The importance values of N-terminal features, C-terminal features, and Interblock features are 6.43%, 8.81%, and 12.37%, separately. The first three important values of 5 types residues of each block is aromatic residues of Interblock (5.05%), non-polar and aliphatic residues of C-terminal (3.13%), and negatively charged residues of N-terminal (3.00%). The D (aspartate) residues composition of N-terminal, as shown in Figures 3C, is the most important SAAC feature for sub-Golgi classification, but the aromatic residues composition frequency features of the Interblock seem even more important (see Table S3).

To sum up the above, the non-polar and aliphatic residues composition, the (aromatic residues) + (non-polar, aliphatic residues) dipeptide and aromatic residues composition between NH_2_-termial and COOH-terminal of protein sequences are three top biological features for distinguishing the sub-Golgi proteins.

### Metrics Comparison With Existing Predictors

Ten optimized sub-Golgi classifiers that have been developed are presented in Table 3. Three separate data sets (D0, D1, D2), and four machine learning algorithms (IDMD, SVM, KNN, RF) were exploited to train these sub-Golgi classifiers, and one common independent data set was used to evaluate the various sub-Golgi classifiers. A total of six classifiers adopted the PSSM and its derived features for sub-Golgi prediction. Ahmad et al. (2017), training on the D2 data set with 40% sequence identity, achieved the highest independent testing scores (ACC = 94.8%; MCC = 0.86; Sn = 93.9%; Sn = 94.0%) for a classifier; the KNN sub-Golgi classifier with 83 composited features. In contrast to the KNN sub-Golgi classifier of Ahmad et al. the ultimate classifier rfGPT_4 in this paper was trained on the benchmark data set D1 with 25% sequence identity and contained 93 features, without any PSSM and its derivative features. Therefore, the rfGPT_4 is more practical, because the time-consuming sequence alignment step to obtain the PSSM and its derivatives scores using the Position-Specific Iterative Basic Local Alignment Search Tool is avoided. In addition, rfGPT_4 is currently the model with the best independent testing scores for training on data set D1 and is a state-of-art sub-Golgi classifier with only dipeptide and amino acid composition features.

**Table 3:** Jackknife cross-validation and independent testing scores list for reported sub-Golgi protein classifiers.

**No**.	**Classifier (Reference)**	**Data Set**	**Features**	**Dim**	**Jackknife cross-validation**				**Independent testing**			
					**ACC**	**MCC**	**Sn**	**Sp**	**ACC**	**MCC**	**Sn**	**Sp**
1	IDMD (Ding et al., 2011)	D0	2-gapDC	400	74.7%	0.495	79.6%	69.6%	/	/	/	/
2	SVM (Ding et al., 2013)	D1	2-gapDC	83	85.4%	0.652	90.5%	90.5%	85.9%	0.578	90.2%	69.2%
3	SVM (Jiao and Du, 2016a)	D1	PSPCP	59	86.9%	0.684	92.6%	73.8%	/	/	90.2%	69.2%
4	SVM (Jiao and Du, 2016b)	D1	PSPCP	49	91.2%	0.793	99.0%	73.8%	87.1%	/	/	/
5	SVM (Lin et al., 2014)	D1	TPDC	501	97.1%	0.949	100%	92.9%	/	/	/	/
6	SVM (Rahman et al., 2018)	D2	ACC +DPDC +TPDC +2-gapDC +PseAAC	2800	95.9%	0.920	95.9%	92.6%	93.8%	0.85	98.0%	84.6%
7	KNN (Ahmad et al., 2017)	D2	PseAAC +3-gapDC +Bigram-PSSM	83	94.9%	0.90	97.2%	92.6%	94.8%	0.86	93.9%	94.0%
8	KNN (Ahmad and Hayat, 2019)	D2	SAAC +PSSM +3-gapDC	180	98.2%	0.96	98.6%	97.7%	94%	0.84	96.9%	81.5%
9	RF (Yang R. et al., 2016)	D2	3-gapDC +CSP-PSSMDC +CSP-BigramPSSM +CSP-EDPSSM	55	88.5%	0.765	88.9%	88%	93.8%	0.821	94.1%	92.3%
10	RF (this work)	D1	2-gapDC+SAAC	93	90.5%	0.811	92.6%	88.4%	90.6%	0.696	96.1%	69.2%

## Conclusions

In this work, an optimized rfGPT classifier for sub-Golgi protein type (cis and trans) identification was developed. The rfGPT classifier was derived from a random forests machine-learning algorithm, followed by implementation of the SMOTE to overcome a severe imbalance in the training data set and selection of optimal-related features using an ANOVA feature selection technique. The independent testing scores (ACC = 90.6%; MCC = 0.696; Sn = 96.1%; Sp = 69.2%) of the rfGPT ranked it as the one of the top sub-Golgi predictors. The feature importance analysis proves that the non-polar and aliphatic residues composition, the (aromatic residues) + (non-polar, aliphatic residues) dipeptide and aromatic residues composition for block between NH_2_-termial and COOH-terminal of protein sequence are the top biological features, which play the key role for sub-Golgi proteins identification.

As compared with previous reported sub-Golgi protein classifiers, the rfGPT is with only dipeptide and amino acid residue composition features, which exempted sequence alignment from the procedure. Also, the rfGPT adopted random forests algorithm is easier for feature analysis and for revealing the key bio-factors of sub-Golgi protein classification. However, the rfGPT had an independent prediction accuracy (from a training data set with 25% sequence identity) that was 4.4% lower than that for the best of the reported sub-Golgi protein identifiers (based on the 40% sequence identity data set) and rfGPT uses more features.

The expectation is to build a more general data set of Golgi protein sequences to train the rfGPT model and to realize a more advanced sub-Golgi classifier of the features. In the future, extreme learning (Li et al., 2019) and deep learning (Long et al., 2017; Yu et al., 2018; Lv et al., 2019; Wei et al., 2019; Zhang Z. et al., 2019; Zou et al., 2019) methods will be tested on this problem.

## Data Availability

Publicly available datasets were analyzed in this study. This data can be found here: http://lin-group.cn/server/subGolgi2.

## Author Contributions

ZL and SJ were responsible for experiments and manuscripts preparation. HD participated in discussions. QZ worked as supervisor for all procedures.

### Conflict of Interest Statement

The authors declare that the research was conducted in the absence of any commercial or financial relationships that could be construed as a potential conflict of interest.

## Abbreviations

D/Dim: dimension
D0/D1/D2/D3: data sets
IDMD: increment diversity Mahalanobis discriminant
SVM: supporting vector machine
KNN: K-nearest neighbors
RF: random forests
2-gapDC: 2-gap dipeptide composition
3-gapDC: 3-gap dipeptide composition
DPDC: Dipeptide compostion
TPDC: Tripeptide composition
AAC: amino acid composition
SAAC: split amino acid composition
PseAAC: pseudo amino acid composition
PSPCP: positional-specific physicochemical properties derived feature from PSSM
PSSM: position-specific scoring matrix
PSSMDC: PSSM-Dipeptide Composition
BigramPSSM: Bi-gram features directly extracted from PSSM
EDPSSM: Evolutionary Difference PSSM
CSP: Common Spatial Patterns
SMOTE: synthetic minority over-sampling technique
ACC: accuracy
MCC: Matthew correlation coefficient
Sn: Sensitivity
Sp: Specificity.
